# Housekeeping from the Higher Standpoint

**Published:** 1911-10-07

**Authors:** Hilda D. Oakeley

**Affiliations:** Warden of King's College for Women.


### INSTITUTIONAL HOUSEKEEPING.

### Housekeeping from the Higher Standpoint.

By Miss HILDA D. OAKELEY, M.A., Warden of King's College for Women.

The claim of the scientific study of housekeeping on the attention and interest of those concerned in hospital work does not perhaps need much urging, and in an introduction of the new department of this paper to its readers I shall only touch upon a single aspect. It seems to me that from one point of view the whole movement for extending and perfecting the education of women in the subjects of home or domestic science may be regarded as an attempt to apply to the whole of life, with a view to the prevention or diminution of disease, the spirit with which we combat disease once it has broken out. Both spheres are dominated by the faith in knowledge—the distinctive ethic of our age, that we are bound to use to the utmost of our power the great forces that knowledge has put into our hands for the well-being of humanity.

### The Hospital Spirit.

The function of the hospital may perhaps be described as that of healing and repairing the human life. The causes why so many men and women fall out of the ranks unable to perform their life-tasks are to be sought largely in some condition of their environment. The hospital, whilst endeavouring to repair the mischief, cannot fail to have a growing insight into the origin. As soon as the weakling of modern society gives up the struggle for which he is so severely handicapped, the hospitals are permitted to take him over and turn upon him the tremendous forces for good at their disposal, in the effort to restore him to the battle in which it is too probable their work will be once more destroyed. But why can science and the higher ethics only step in here? Why may these powers not be earlier and more thoroughly applied to the conditions of life outside the hospital? This thought must often haunt the young doctor and nurse in those efforts, deliberate or involuntary, which we cannot avoid making to form a rude philosophy of our profession.

Outside the hospital the application of science to the amelioration of life is, indeed, being attempted in many directions, but nowhere else with so much perfectness. This is partly, no doubt, because from the nature of the case under no other conditions can effort be so specialised, partly perhaps because the Christian maxim has been too exclusively applied here, "They that are whole need not a physician, but they that are sick." But to the physician observing the sick it must often be painfully evident how much the correction of some insanitary mode of life, habit, or diet might have prevented the fatal change from the whole to the sick state. Before the worker or observer in the sick ward there must sometimes arise the vision of a healthier way of progress over the waters of life in which not so many of those who gaily trim their sails in the early dawn would so soon suffer the shipwreck which has brought them there. Now we are not so bold as to assert that this ideal, this dream of the hospital worker will be fully realised through the better application of science to the apparatus of home life—using home in the widest sense, as the background of human existence, in which it renews its forces day by day. It may, however, at least be said that the present movement in this direction has a very important place in the warfare with disease and physical disability at every stage, the construction of a world in which diseases of the body and the mind will be far less frequent.

### Pressing Problems of the Moment.

The power of knowledge is not felt outside with the same conviction that it is within the hospital. The modern reformer is often more ready to trust in the power of wealth, of material resources, to combat social and physical maladies. But it is not only where these resources are lacking that we meet the troubles which arise from imperfect conditions of living. The most pressing, most universally needed step towards their improvement is a wiser, more thorough use of the existing forces of science, together with that more zealous, more intelligent study and search which will increase those forces. The men and women who go about their houses and the streets of their cities, the offices where they work, the institutions, factories, business places where they employ or co-operate with others, ignorant of the causes why their way of life, their arrangement of their scenes of work or pleasure, the occupations they take up, or to which they set others, are healthy or unhealthy, are *pro tanto* blind in a world that ought to be well-lighted for them. The light is in the charge of science and ready to stream on them if they will but ask for it. If they do not even realise the need, they have what Plato called falseness or darkness in their souls. This is, in the first place, a practical danger, but it is also, as we are trying to make the educational world understand, a lack in true culture.

It is not necessary, however, to argue in these pages that there can be in this matter no sharp line drawn between the ideal and the utilitarian. For these two aspects, which are indeed everywhere intermingled in human life, are so in a supreme degree in the hospital, and no one can deter the doctor or the nurse from their incomparably useful vocation by the criticism that it is utilitarian or wanting in idealism. It is, therefore, that it is a consolation and an encouragement to those of us who are advocating the admission of the studies described as home science to a high place in education, and who are frequently met by the objection that the subject is too utilitarian, to think of ourselves as in some respects—if at a distance—fellow-workers with those engaged in the profession of healing.

